# Medical Characteristics of Foreign Language Patients in Paramedic Care

**DOI:** 10.3390/ijerph17176306

**Published:** 2020-08-30

**Authors:** Frank Müller, Eva Hummers, Eva Maria Noack

**Affiliations:** Department of General Practice, University Medical Center Göttingen, 37073 Göttingen, Germany; eva.hummers@med.uni-goettingen.de (E.H.); evamaria.noack@med.uni-goettingen.de (E.M.N.)

**Keywords:** language barrier, migrant, refugee, suicide attempt, birth, emergency medical service

## Abstract

Background: In this study, medical and socio-demographic characteristics of foreign language patients in prehospital emergency medical care are analyzed and compared with non-foreign language patients. Methods: We performed retrospective chart review of rescue operations in four emergency medical service stations in Northern Germany over seven months as part of the DICTUM Rescue study (DRKS00016719). We performed descriptive analyses including test statistics and used partial correlation to adjust for patients’ sex and age. Results: Patients with limited German proficiency were served in 2.2% of all 7494 covered rescue operations. On average, these patients were two decades younger than their German speaking counterparts. There were significantly more patients with limited German proficiency with gynecological and obstetric problems, especially births, as well as psychiatric disorders, especially suicide attempts. Conclusions: Our findings suggest that the existing preventive programs for pregnant women and people at risk of suicide do not sufficiently reach patients with limited German proficiency. Additionally, giving birth and psychiatric breakdowns are exceptional and sensitive situations, both for patients and the paramedic staff, where the ability to communicate safely appears to be of enormous importance to enable safe treatment.

## 1. Introduction

In recent decades, the ethnic heterogeneity of people living in Germany has increased. In addition to the more than 2.3 million refugees seeking protection in the last five years [1,2], Germany is a destination for workers from all over Europe as well as for tourists and business travelers from all over the world [3]. As a result, medical professionals are increasingly caring for people who speak little or no German and with whom communication is therefore challenging.

Challenges arise particularly in the pre-hospital emergency care provided by emergency medical services (EMS). In these situations, the use of professional interpreters, which are the gold standard for overcoming language barriers, may not be present in medical emergency situations for many reasons. Other options such as video interpreters are difficult to implement in the time-critical EMS sector, as they may delay necessary transport to hospital. As a consequence, paramedics have to perform an initial assessment completely without interpreters, or sometimes they have to rely on the assistance of lay interpreters, often family members, or try to communicate in a third language, unless paramedics happen to speak the patient’s language [4,5]. It can be assumed that information relevant for the treatment of patients with limited proficiency of the locally spoken language, such as patients’ medical history and medication, can only be collected with difficulty and high uncertainty. Furthermore, research shows that language discordance is a barrier to use EMS [6,7,8]. Foreign language speaking patients were uncertain when to call for EMS [9]. Various studies show that dispatching was delayed or inaccurate when there was a language barrier [10]. It also appears that resources (advanced life support vs. basic life support) were distributed differently if the caller was not proficient in the local language [5], e.g., there was an increased dispatch of advanced life support trained paramedics [11]. All these findings strongly suggest that language skills in the local language are essential to receive adequate and safe medical treatment by EMS in case of the occurrence of a medical emergency. It is still unclear how language barriers affect the quality of preclinical emergency care and patient-relevant outcomes [12,13]. Little information is available about the medical conditions with which foreign-language patients seek emergency medical help. Given the demographic differences, for example, between migrants or refugees and the autochthonous population, relevant differences seem likely. Identifying and understanding these differences is necessary to ensure high-quality care for this group of patients. As part of the DICTUM rescue study, where we develop app-based technologies to overcome language barriers in EMS, we conducted a chart review of rescue operations to determine the medical characteristics of the patients [14].

## 2. Materials and Methods

We conducted a retrospective chart review of depersonalized EMS cases of four EMS stations over seven months (15 May 2019–15 December 2019). In Germany, every EMS operation is documented on a standardized protocol. Sociodemographic characteristics of patients (age, gender), their current medical conditions, working diagnoses, the paramedics’ description of the emergency case (free text), the need for ventilation, and data on the rescue operation (time en route to reach patient, time spent on emergency scene) were extracted from this protocol. Data extraction from paper-based protocols for the Brunswick EMS Station was performed by an experienced study nurse. The other EMS stations use a digital protocol software (CEUS^®^ Rettungsdienst, CKS Systeme GmbH, Meppen, Germany) for documenting, thus we exported data for analyses. Patients with limited German language proficiency (LGP) and their primarily spoken language were identified manually analyzing each free text comment made by paramedics on each case. We excluded all cases of hospital to hospital transport and rescue operations with no patient present or no patient served on the emergency scene. In total, we collected information on 7494 rescue operations.

**Figure 1:**
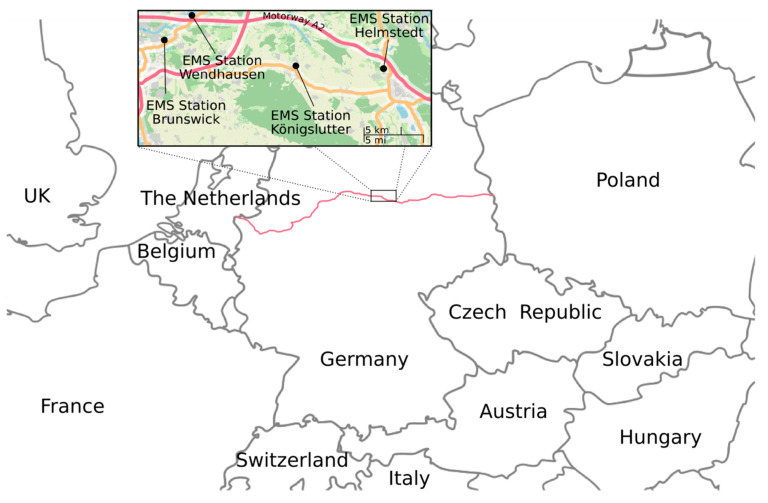
Locations of the recruiting rescue service stations in Germany. The red line shows the course of the A2 motorway, one of the most important east-west transport corridors between Eastern Europe and the Dutch seaports.

We used SPSS (v25, IBM, Armonk, NY, USA) for statistical analyses. Figures were constructed using Google Spreadsheet (Alphabet Inc., Mountain View, CA, USA). The map used in Figure 1 is based on material of OpenStreetMap (www.openstreetmap.org, published under Creative Commons Attribution (CC-BY-SA 2.0), ©OpenStreetMap contributors). To describe our sample, we used absolute and relative frequencies as well as mean values, standard deviations (SD), median, and interquartile range (IQR). Chi-square and Fisher’s exact test were used to test categorical variables and Mann-Whitney *U* test for testing metric and categorical variables. Associations were also estimated using partial correlation adjusting for age and sex. *p*-values less than 0.05 were considered as significant.

The Research Ethics Board of the University Medical Center Göttingen provided approval for the study to be conducted on all trial sites (Approval No. 9/9/18). The study was registered at the German Clinical Trials Register (No. DRKS00016719). We concluded cooperation agreements with the EMS providers participating in this study as well as with the Municipality of Brunswick and the District of Helmstedt.

We conducted our study in Northern Germany in the District of Helmstedt (approx. 91,000 inhabitants) and the Municipality of Brunswick (approx. 248,000 inhabitants). The EMS stations Königslutter, Wendhausen, and Helmstedt are located in a rather structurally weak rural region adjoining the former inner-German border, whereas the EMS station in Brunswick is located in the second largest city in Lower Saxony. The rescue service stations Wendhausen and Helmstedt are located close to the motorway A2, which is the most important east-west route in northern Germany, connecting the Netherlands with its significant harbors with Poland and other countries in the region of north-eastern Europe. The location of the EMS stations is shown in Figure 1. The LGP patients treated by the EMS include migrants and refugees living regularly in Germany as well as transit travelers and seasonal workers. A reliable differentiation of these groups is not possible in the study, as this aspect is usually not documented in the EMS operation protocols. Additionally, due to the variety of different mobility and (working-)migration practices, especially within the European Union, an approach that follows a further differentiation seems unfeasible.

The use of EMS is generally covered by the statutory health insurance and is therefore free of charge. This also applies to asylum seekers. For most citizens of the European Union and some other countries such as Serbia, Switzerland, or Norway, any costs for emergency medical services are covered within the framework of the European Health Insurance Card (EHIC).

## 3. Results

**Figure 2:**
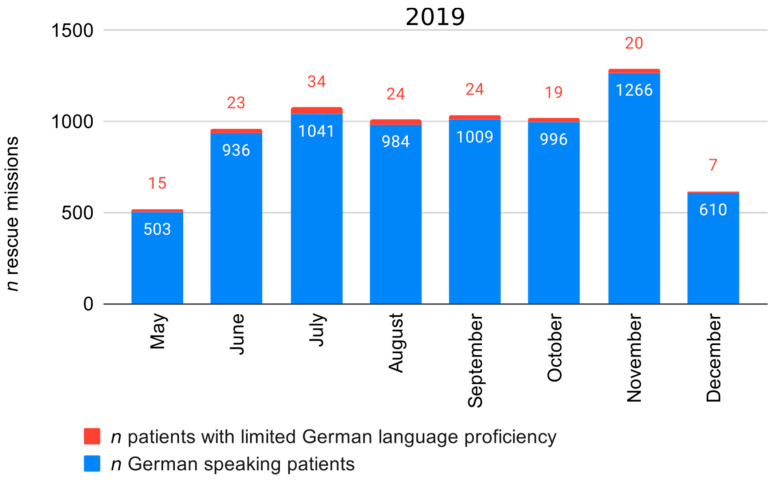
Rescue operations during the study period in 2019.

In total, we covered 7494 EMS rescue operations. Nearly one third was carried out by the urban EMS station in the City of Brunswick (*n* = 2326, 31.0%), followed by the rural EMS stations Wendhausen (*n* = 1960, 26.2%), Helmstedt (*n* = 1776, 23.7%), and Königslutter (*n* = 1432, 19.1%). Rescue operations were nearly equally distributed over the course of the seven months and amounted to around 1000 operations per month (Figure 2). A slightly increased frequency was detected in November 2019 with a total of 1286 rescue operations. One third (33.3%, *n* = 1830) of all rescue operations were supplemented by an emergency physician. It took the paramedics on average 8.5 min (SD 4.9 min) to reach the patient. In 3.5% (*n* = 260) of all rescue operations, patients rejected care and transport to destination.

Transported patients were in average 61.4 years old (SD 24.8 years) and were mostly affected from symptoms regarding the cardiovascular system (25.4%) followed by neurological disorders (11.1%). The mean Glasgow Coma Scale (GCS) Score was 14.1 (SD 2.6), and just a fraction of patients required invasive ventilation (*n* = 102, 1.4%).

### EMS Operations with Patients with Limited German Language Proficiency

**Figure 3:**
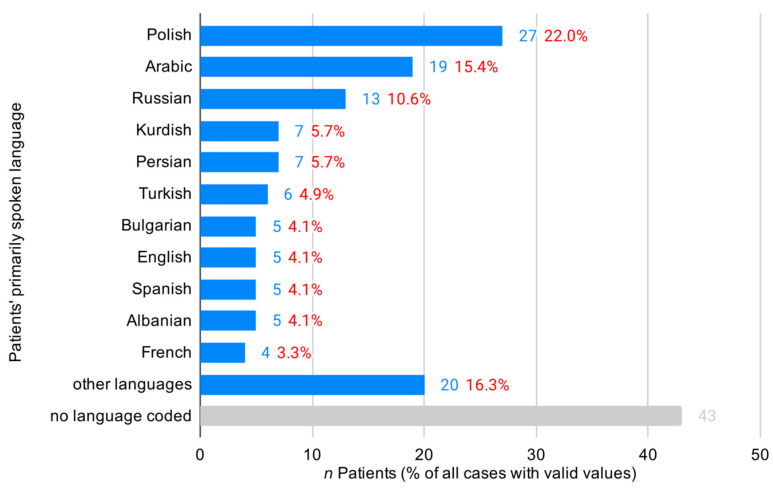
Distribution of patients’ primarily spoken language.

In total, we identified rescue operations with 166 patients (2.2%) with limited German language proficiency. With a mean age of 39.2 years (SD 20.8), these patients were on average over two decades younger than the German speaking patient (GSP) group (*p* < 0.001). In total, 12.6% of LGP patients were underage children vs. 6.7% in the GSP group (X^2^ = 8.305, df = 1, *p* = 0.005), and 55.8% of LGP patients were male compared to 51.9% of GSP (Table 1). The most common language spoken by LGP patients was Polish (22.0%), followed by Arabic (15.4%) and Russian (10.6%) (Figure 3).

LGP patients and German speaking counterparts did not differ regarding the additional dispatch of emergency physicians (33.5% vs. 26.7% in LGP patients, *p* = 0.119). Moreover, no differences were observed in the timespans it took paramedics to reach the patient (mean 8.5 vs. 8.5 min in LGP patients, *p* = 0.908), the time spent on scene (mean 21.7 vs. 20.2 min in LGP patients, *p* = 0.280), or the time to transport patients to destination (mean 15.9 min vs. 16.0 min in LGP patients, *p* = 0.426) (Table 2).

Patients with limited German language proficiency were mainly affected by cardiovascular problems (*n* = 36, 23.4%), psychiatric disorders (*n* = 19, 13.0%), and neurological ailments (*n* = 17, 11.0%). In comparison with German speaking patients, psychiatric disorders were more frequent in the LGP group (13.0% vs. 8.6%, *p* = 0.048), but this finding did not remain significant when adjusted for age and sex (*p* = 0.871). Gynecological and obstetric problems among patients with LGP were clearly more frequent than in GSP (6.5% vs. 1.1%, *p* < 0.001). This finding remained significant when adjusted for age (*p* < 0.001). Rescue operations with injured LGP patients occurred less frequently than with GSP (18.8% vs. 24.3%, *p* = 0.007 (age and sex adjusted)). Especially moderate (*n* = 6) and severe injuries (*n* = 0) were observed less often in LGP patients. LGP patients had a slightly but significantly higher GCS (mean value 14.5 vs. 14.1, *p* = 0.007) but not when adjusted for age and sex. More information is provided in Table 3.

A more detailed analysis of symptoms and diseases revealed that rescue operations with imminent childbirth (4.5% (*n* = 7) vs. 0.5% (*n* = 33), *p* < 0.001, adjusted by age: *p* < 0.001) as well as suicide attempts (4.5% (*n* = 7) vs. 0.5% (*n* = 30), *p* < 0.001, adjusted by age and sex: *p* < 0.001) were observed significantly more frequently among LGP patients. An analysis of the free text fields showed that, in six of the seven childbirth cases, the week of pregnancy could be determined (all were between 34th and 40th week of pregnancy). In four cases, the women had labor activity with intervals of 5 min or less, and in two cases, no labor activity was observed. In four of the seven women, it was noted that they had previously delivered three or four children. The languages spoken by the women giving birth suggest that they are permanently living in Germany as migrants or refugees. In contrast, for the German speaking mothers, it was mainly the first (*n* = 7, 63.6%) or the second delivery (*n* = 4, 34.4%). The mothers with LGP were between 19 and 38 years old (mean 27.29, SD 7.48) and thus on average four years younger than German speaking mothers (mean age 31.55 years, SD 5.09, min 17 years, max 39 years, *p* = 0.158). No relevant previous illnesses or complications during transport were described for LGP patients.

All seven suicidal patients with LGP were men between 19 and 60 years of age (mean 33.14, SD 14.62). In contrast, GSP with suicidal behavior were on average six years older (mean 39.40 years, SD 18.80, min 15 years, max 69 years, *p* = 0.614), and 41.4% of patients were female (*n* = 12). The sex distribution differed significantly between LGP and German speaking suicidal patients (*p* = 0.037). Three LGP patients tried to commit suicide by overdosing medication and were found somnolent by the paramedics. Three patients were acutely agitated. Three patients were living in a refugee accommodation; in two other cases, the patients were encountered in ambulant psychiatric care facilities.

## 4. Discussion

To the best of our knowledge, our study is the first ever that examines the medical characteristics of foreign language patients in prehospital emergency medical care. In a total of 7949 rescue operations, we were able to identify 166 (2.2%) patients who spoke little or no German. These patients were more than two decades younger than their German counterparts and mostly spoke languages from Eastern Europe or the Middle East. The proportion of underage children in the LGP patient group was twice as high as among German speaking patients. This finding needs further investigation; however, due to the low absolute numbers of underage LGP patients (*n* = 20), we were not able to perform a more detailed analysis. This age difference can be explained by the generally lower age of migrants [1] and has also been shown in another study [15]. However, our findings are contrary to those of Weiss et al. who report more female and older patients with limited English proficiency in a study in the United States [16].

In terms of the rescue operation characteristics, i.e., the time it took the paramedics to reach the patient, there was no significant difference between the analyzed groups. This could indicate that, despite language barriers, patients or bystanders who call the emergency medical services can make themselves sufficiently clear about the location of the incident, and no time is lost by paramedics having difficulties finding the patients. However, due to the study design, this cannot be said with certainty, as the time to reach the patient also depends on a number of other factors, such as distance or traffic congestions, which could not be considered in the study. The average time paramedics spent on scene was about one minute shorter with LGP patients, however, this finding was not significant. This trend was also described by other researchers [10,17].

LGP patients differed in terms of initial assessment and initial diagnosis. There were significantly more patients with gynecological or obstetric needs, especially spontaneous births. A study carried out in North German initial reception centers for asylum seekers and refugees showed that 9.1% of all women of child-bearing age coming to Germany were pregnant [18]. Normally, women in Germany are accompanied by community midwives during pregnancy and after giving birth. All maternity medical care is covered by the statutory health insurance and is also free of charge for refugee patients. Women may attend birthing classes and are instructed by midwives about the birth process and infant care. Typically, parents-to-be decide on a hospital or an outpatient birth center where they want to give birth months before the estimated date of birth. When a pregnant woman then goes into labor, she independently visits her designated hospital or birth center. The increased use of EMS by LGP patients permanently living in Germany as refugees or migrants may therefore imply that pregnancy care of this group should be improved, especially with regard to navigation through the health care system.

In our study, the increased number of suicide attempts in LGP group was striking, and the overall incidence of psychiatric disorders was higher among LGP patients but not significant when adjusted for age and sex. This finding seems less surprising in view of the fact that migrants and refugees are more likely to suffer from psychiatric disorders such as posttraumatic stress disorder (PTSD) and depression [19,20,21]. This finding may suggest that suicide prevention programs do not sufficiently reach male LGP patients in particular. Furthermore, it implies that EMS staff are more likely to encounter suicidal LGP patients, which should be taken into account in EMS training programs. Additionally, psychiatric issues among LGP patients might have been underestimated, as the psychiatric status was often not assessed in this patient group. This may have to do with the fact that an assessment is very difficult due to the language barrier.

The present study is subject to some limitations that should be considered when interpreting the results. For example, the LGP cases were identified using a corresponding note by paramedics in the free text field. As a result, a certain number of LGP cases were probably not identified, for example, when communication worked sufficiently well (for example, by using English as a third language) or may not have played a relevant role in the patient’s care. This is particularly applicable to patients who were seriously ill, so that immediate medical assistance was required, or when injuries and circumstances were obvious and did not need a further investigation. Accordingly, there were no patients in the LGP group that required assisted ventilation, had serious injuries, and only one patient needed resuscitation. This is also reflected in the fact that the GCS score was significantly higher than in the German language speaking group. It is also possible that language barriers were not recorded if paramedics happened to have language skills of the patient’s preferred language. We did not systematically investigated EMS staff’s language skills; however, to our knowledge, very few multilingual EMS staff or paramedics with a migrant background are employed in the surveyed EMS stations. Therefore, our study reflects the paramedics’ perspective on patients with language barriers in the rescue service rather than providing valid data on the disease distribution of LGP patients. We were also not able to take special characteristics of patients such as the primarily spoken language into account, and only selected subgroups could be studied separately due to the sample size. Likewise, we could only gather limited data for certain conditions of LGP patients. However, the differences outlined here call for further investigation, also considering the perspectives of both paramedics and LGP patients about how emergency rescue services are provided and experienced.

## 5. Conclusions

Our study has shown that the reasons to call emergency medical service diverge between German speaking patients and foreign-language patients. These differences can partly be explained by the demographic characteristics of patients with LGP. Paramedics are often overly challenged with providing care to suicidal (male) LGP patients and LGP patients giving birth. Language barriers appear to be particularly problematic in such exceptional situations, as they can adversely affect patient outcomes and the safety of paramedic staff.

However, the principal and structural obstacles that arise due to language barriers in the general field of EMS remain and call for both further investigation and interventions. These interventions could address a variety of aspects, e.g., by enhancing patients’ knowledge about when and how to call for an EMS, by increasing cultural and language sensitivity of paramedic staff, by promoting greater ethnic and linguistic diversity in the EMS field, or by introducing feasibly app-based digital solutions that help paramedics to communicate with foreign language patients.

## Figures and Tables

**Table 1:** Patients’ demographics.

Rescue Operations		Unit	All (*n* = 7494)	GSP (*n* = 7328)	LGP Patients (*n* = 166)	*n* Missing	*p*
Sex	Male	*n* (%)	3531 (52.0)	3444 (51.9)	87 (55.8)	706	0.571
	Female	*n* (%)	3246 (47.8)	3177 (47.9)	69 (44.2)		
	Other	*n* (%)	11 (0.2)	11 (0.2)	0 (0.0)		
Age		years; mean (SD)	61.4 (24.8)	61.9 (24.7)	39.1 (20.6)	532	<0.001
Children (<18 years)		*n* (%)	478 (6.4)	458 (6.7)	20 (12.4)	532	0.005

GSP: German speaking patient; LGP: limited German language proficiency.

**Table 2:** Characteristics of rescue operation.

Rescue Operations	Unit	All (*n* = 7494)	GSP (*n* = 7328)	LGP Patients (*n* = 166)	*n* Missing	*p*	*P* (Adjusted *)
Time en route (min)	Mean (SD)	8.5 (4.9)	8.5 (4.9)	8.5 (4.7)	893	0.908	0.816
	Median (IQR)	7 (6)	7 (6)	8 (6)			
Time on scene (min)	Mean (SD)	21.6 (12.2)	21.7 (12.3)	20.2 (10.3)	1780	0.280	0.901
	Median (IQR)	19 (13)	19 (14)	18.5 (13)			
Time to destination (min)	Mean (SD)	15.9 (9.3)	15.9 (9.4)	16.0 (8.1)	2121	0.426	0.983
	Median (IQR)	15 (9)	15 (9)	17 (11)			
Dispatch at nighttime (20:00–07:00)	*n* (%)	2527 (34.7)	2469 (34.7)	59 (36.2)	219	0.692	0.992
Emergency physician present	*n* (%)	1830 (33.3)	1798 (33.5)	32 (26.7)	1999	0.119	0.134
Rural area	*n* (%)	5168 (69.0)	5052 (68.9)	116 (69.9)	0	0.796	0.601
Patient rejected care	*n* (%)	260 (3.5)	257 (3.5)	3 (1.8)	0	0.237	0.105

(*) Partial correlation adjusting for patients’ sex and age.

**Table 3:** Medical characteristics of patients.

Rescue Operations		Unit	All (*n* = 7494)	GSP (*n* = 7328)	LGP Patients (*n* = 166)	*n* Missing	*p*	*p* (Adjusted ^*^)
**Initial Assessment**								
Glasgow Coma Scale (GCS)		Mean (SD)	14.1 (2.6)	14.1 (2.6)	14.5 (2.1)	597	0.012	0.176
NACA score ^#^		Mean (SD)	3.1 (1.3)	3.1 (1.3)	3.1 (1.2)	4170	0.852	0.291
Psychiatric symptoms		*n* (%)	789 (12.4)	779 (12.5)	10 (6.5)	1128	0.034	0.519
Need for ventilation		*n* (%)	102 (1.4)	102 (1.4)	0 (0.0)	0	0.126	0.216
**Preliminary Diagnosis**								
Neurological disorders		*n* (%)	729 (11.1)	712 (11.1)	17 (11.0)	907	0.991	0.850
Cardiovascular disorders		*n* (%)	1673 (25.4)	1637 (25.4)	36 (23.4)	907	0.560	0.051
Respiratory disorders		*n* (%)	592 (9.0)	584 (9.1)	8 (5.2)	907	0.096	0.214
Metabolic disorders		*n* (%)	427 (6.5)	422 (6.6)	5 (3.2)	907	0.099	0.854
Psychiatric disorders		*n* (%)	565 (8.6)	546 (8.5)	19 (13.0)	907	0.048	0.871
Abdominal disorders		*n* (%)	662 (10.1)	646 (10.0)	16 (10.4)	907	0.887	0.646
Gynecological and obstetric disorders		*n* (%)	79 (1.2)	69 (1.1)	10 (6.5)	907	<0.001	<0.001
Other disorders		*n* (%)	586 (8.9)	573 (8.9)	13 (8.4)	907	0.841	0.868
Injuries	None	*n* (%)	5902 (75.8)	5767 (75.7)	135 (81.2)	907	0.117	0.007
	Slight	*n* (%)	1037 (15.7)	1014 (15.8)	23 (14.9)	907	0.781	0.153
	moderate	*n* (%)	493 (7.5)	487 (7.6)	6 (3.9)	907	0.087	0.065
	Severe	*n* (%)	62 (0.9)	62 (1.0)	0 (0.0)	907	0.505	0.157

^(*)^ Partial correlation adjusting for patients’ sex (except gynecological and obstetric disorders) and age. (**^#^**) National Advisory Committee for Aeronautics Score.
